# Comparison of structural occipital and iliac bone grafts for instrumented atlantoaxial fusions in pediatric patients: Radiologic research and clinical outcomes

**DOI:** 10.3389/fsurg.2023.1059544

**Published:** 2023-03-21

**Authors:** Zhi-Hui Liang, Yue-Hui Zhang, Hai-Tao Liu, Qiu-Qi Zhang, Jia Song, Jiang Shao

**Affiliations:** Spine Center, Xin Hua Hospital Affiliated to Shanghai Jiao Tong University School of Medicine, Shanghai, China

**Keywords:** atlantoaxial fusion, iliac bone graft, occipital bone graft, structural autograft, atlantoaxial dislocation

## Abstract

**Background:**

Structural autografts harvested from the iliac bone have been used in atlantoaxial fusion; they have been the gold standard for years. However, emerging occipital bone grafts have the advantage of avoiding donor-site morbidity and complications. Thus, we compared the clinical outcomes of structural autografts from the occipital bone or iliac crest and discussed the clinical significance of occipital bone grafts in pediatric patients.

**Methods:**

Pediatric patients who underwent posterior fusion using occipital bone grafts (OBG) or iliac bone grafts (IBG) between 2017 and 2021 were included in this study. Data on clinical outcomes, including operation time, estimated blood loss, length of hospitalization, complications, fusion rate, and fusion time, were collected and analyzed. Additionally, 300 pediatric patients who underwent cranial computed tomography scans were included in the bone thickness evaluation procedure. The central and edge thicknesses of the harvested areas were recorded and analyzed.

**Results:**

Thirty-nine patients were included in this study. There were no significant differences in patient characteristics between the OBG and IBG groups. Patients in both groups achieved a 100% fusion rate; however, the fusion time in the OBG group was significantly longer than that in the IBG group. Estimated blood loss, operation time, and length of hospitalization were significantly lower in the OBG group than those in the IBG group. The surgery-related complication rate was lower, but not significantly, in the OBG group than that in the IBG group. For occipital bone thickness evaluation, a significant difference in the central part of the harvesting area was found between the young and old groups, with no significant sex differences.

**Conclusion:**

The use of OBG for atlantoaxial fusion is acceptable for pediatric patients with atlantoaxial dislocation, avoiding donor-site morbidity and complications.

## 1. Introduction

Atlantoaxial dislocation is defined as a loss of normal articulation and instability of the atlantoaxial joint, often resulting from trauma and congenital and inflammatory factors (1, 2). The surgical treatment of atlantoaxial dislocation is focused on decompressing the spinal cord and reconstructing a stable atlantoaxial joint (3–5). Atlantoaxial fusion can regenerate stable joint constructs and promote solid arthrodesis, and a bone graft is a key factor in the fusion process (6). Recently, cadaveric allografts for pediatric atlantoaxial fusion showed their competence with lower blood loss, shorter operative time, and no donor-site morbidity (7); however, they have the risk of infectious transmission and a relatively low fusion rate (5, 8). Furthermore, in pediatric upper cervical spine fusion, the use of iliac bone grafts (IBG) was considered the gold standard due to their high fusion rate. However, the potential donor-site complications, prolonged operation time, and increased estimated blood loss resulting from the second incision need to be seriously considered (8).

An occipital bone graft (OBG) is an ideal material for spine fusion consisting of bicortical elements and a diploe, ensuring a rich blood supply and biomechanical strength for the graft. It has the advantages of IBG and allografts, with a relatively high fusion rate and no requirement for an extra incision (9). Previous studies found that an OBG, as a membranous bone, has the advantage of less resorption than an IBG or rib, which are endochondral bones (10, 11). Several studies have reported the use of OBG for atlantoaxial fusion in adult and pediatric patients (9, 12–14); however, no research has compared the clinical outcomes of OBG and IBG with the use of the screw/rod fixation technique in pediatric patients and evaluated the thickness of the occipital bone donor site for safety concerns. Therefore, the main purpose of this study was to evaluate the clinical outcomes of OBG and IBG with the use of rigid screw/rod fixation techniques in pediatric patients.

## 2. Methods

The study protocol was approved by the Ethics Committee of Xinhua Hospital (XHEC-D-2022-193). This study followed the principles of the Declaration of Helsinki and the laws and regulations of the People's Republic of China, and all patients provided written informed consent.

**Figure 1:**
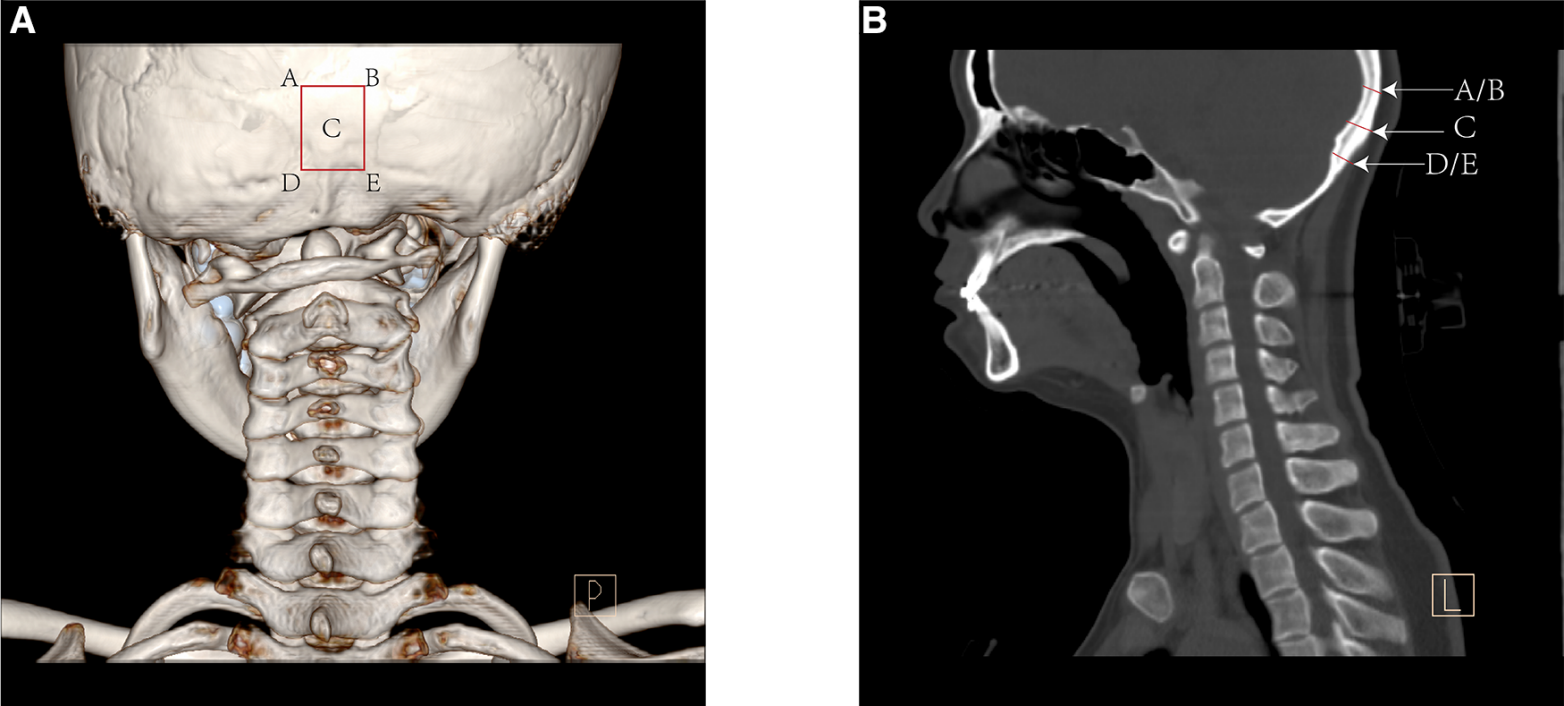
The posterior view of computed tomography (CT) 3D reconstruction (**A**) and the sagittal reconstruction (**B**) showed the method to measure the bone thickness of the occipital donor-site.

The small population of pediatric patients with atlantoaxial dislocation provided limited information to evaluate the thickness of the occipital bone donor site. Hence, we included 300 pediatric patients aged 2–12 years who underwent computed tomography (CT) scanning for concussion or brain damage and the scanning area included the occipital bone. The center of the occipital donor site was defined as the external occipital protuberance. Additionally, the thickness of the four corners in the 1.5 cm × 2 cm donor site was measured, and the average thickness of the four corners was considered the edge thickness (Figure 1).

For atlantoaxial fusion evaluation, 39 pediatric patients who underwent atlantoaxial fusion for atlantoaxial dislocation with OBG and IBG in the authors' hospital between 2017 and 2021 were retrospectively evaluated and analyzed. Age, sex, body weight, diagnosis, graft type, operation time, estimated blood loss, approach, type of instrumentation, length of hospitalization, postoperative follow-up period, fusion time, intra-and perioperative complications, and donor-site morbidities were examined. Patients diagnosed with tumors or serious infections were excluded. Surgical indications included neurological deficits, neck pain or occipital headache, and limitation of neck motion, with or without severe spinal cord compression.

### 2.1. Surgical procedure and clinical follow-up

The patients were placed under general anesthesia, and cranial skeletal traction was applied. Irreducibility was determined if the patient did not show a satisfactory reduction under skeletal traction. For irreducible atlantoaxial dislocation, anterior release through the retropharyngeal approach described in a previous study was performed (15).

After satisfactory reduction was achieved, the patient was placed in the prone position for posterior instrumentation and fusion. After exposure of the posterior side of C1–C2, C1 pedicle screws were inserted as previously described (16–18). Briefly, the vertebral artery was cranially dissected away from the posterior arch of the atlas, while the C2 root was caudally dissected away. The vertebral artery and C2 root were protected using two Penfield dissectors. The pilot hole was drilled and deepened as previously described (5). The C1 polyaxial screws were inserted through the pilot holes, and C2 pedicle screws were placed as described by Harms et al. (19); after satisfactory reduction was confirmed using fluoroscopy, titanium fixation rods were placed.

The midline incision was extended over the external occipital protuberance for the OBG harvesting procedure. A 1.5 cm × 2 cm split-thickness OBG was obtained using piezosurgery or an osteotome. For iliac crest bone harvesting, we used the strategy of harvesting a 1.5 cm × 2 cm bone graft under the ilium edge to avoid growth plate injury and preserve the integrity of the ilium in pediatric patients. Briefly, a rectangular bone graft was harvested by piezosurgery or osteotome under the edge of the posterior superior iliac spine, bone wax was used to control bleeding. The posterior arch of the atlas and the spinous process of the axis were decorticated using a high-speed bur, and all bone fragments were collected and planted between the bone graft and transplantation bed. The bone graft was trimmed to adapt to the shape of the transplantation bed and then placed and fixed by using wire, as previously described (5, 20). For clinical follow-up, the Philadelphia collar would be placed for 3 months, and CT scans were conducted 6 months postoperatively, and the interval between CT scans was 3 months until osseous fusion was achieved. A three-dimensional reconstructive CT scan was used to identify complete fusion.

### 2.2. Statistical analysis

Statistical analysis was conducted in R, version 4.0.3 (Boston, MA). Values are expressed as mean ± standard deviation. Estimated blood loss, operative time, length of hospitalization, and fusion time of the two groups were assessed using the student's *t*-test or Mann–Whitney test. Fisher's exact test was used to compare fusion and complication rates between the two groups. A *P* value <0.05 was considered statistically significant.

## 3. Results

The occipital bone thickness was measured in 300 pediatric patients, divided into 2–6 and 7–12-year age groups. The results are shown in Table 1. The central thickness of the harvesting area in the male groups of 2–6 and 7–12-year categories was 7.9 ± 1.1 mm and 11.0 ± 1.9 mm, respectively; and in females, the values were 7.3 ± 0.7 mm and 10.0 ± 2.3 mm, respectively. Furthermore, the edge thickness of the OBG in the male groups of 2–6 and 7–12-year categories were 4.8 ± 1.9 mm and 5.1 ± 1.0 mm, respectively, and those in the female group were 4.5 ± 1.0 mm and 5.5 ± 1.9 mm, respectively.

**Table 1:** Occipital bone donor site thickness (mm).

	2–6 years/male	7–12 years/male	*P* value	2–6 years/female	7–12 years/female	*P* value
central	7.9 ± 1.1	11.0 ± 1.9	0.0001	7.3 ± 0.7	10.0 ± 2.3	0.0001
edge	4.8 ± 0.9	5.1 ± 1.0	0.2002	4.5 ± 1.0	5.5 ± 0.9	0.0001

**Figure 2:**
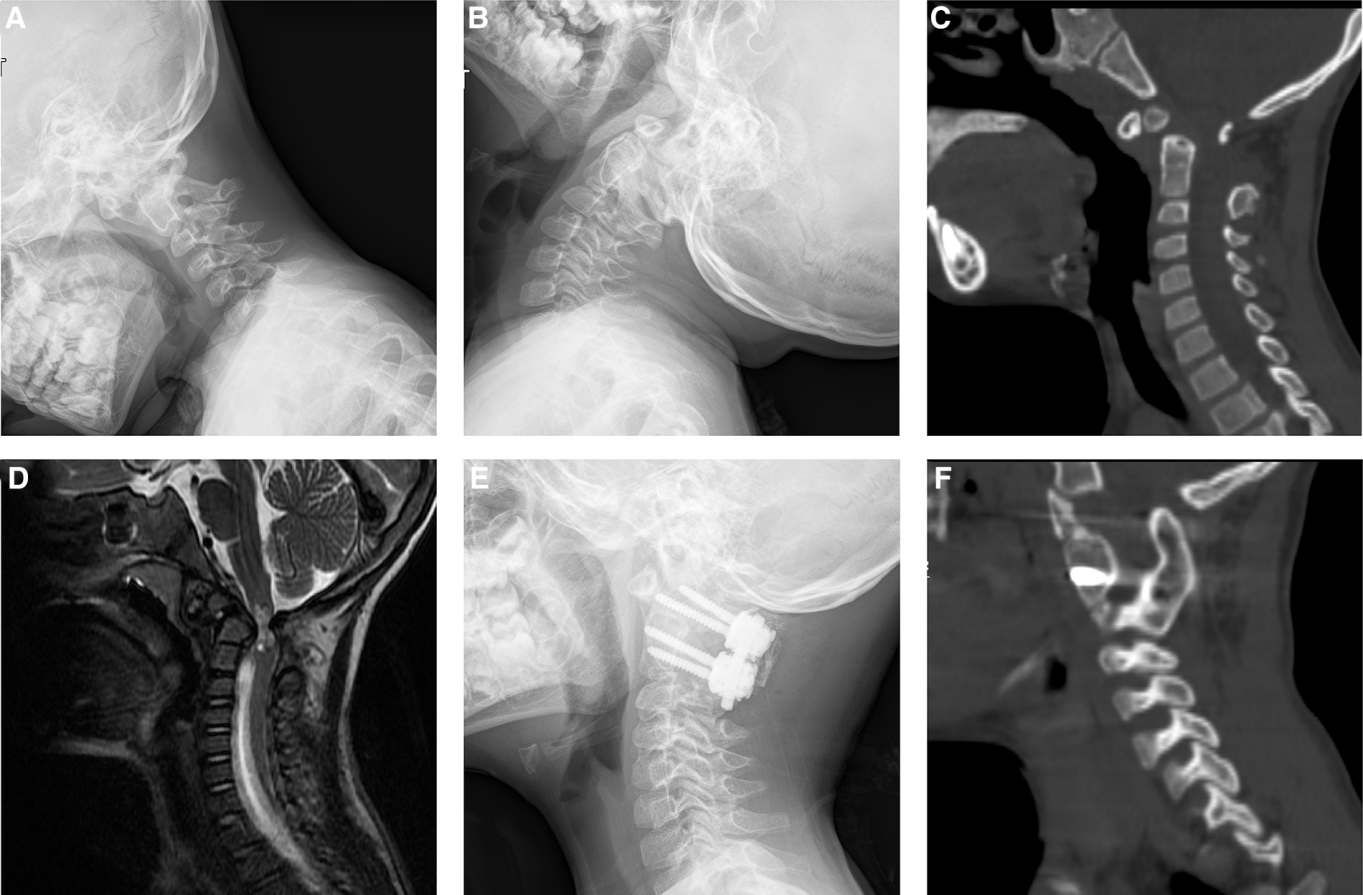
Images of a 5-year-old boy diagnosed of os odontoideum and underwent atlantoaxial fusion with the use of IBG. Preoperative plain radiograph of flexion (**A**) extension (**B**) showed atlantoaxial dislocation. Preoperative CT showed os odontoideum (**C**). Preoperative magnetic resonance imaging (MRI) showed spinal cord compression (**D**). Postoperative plain radiograph (**E**) showed the rigid screw/rod fixation. CT sagittal reconstruction showed solid bony fusion 6 months after surgery (**F**).

**Figure 3:**
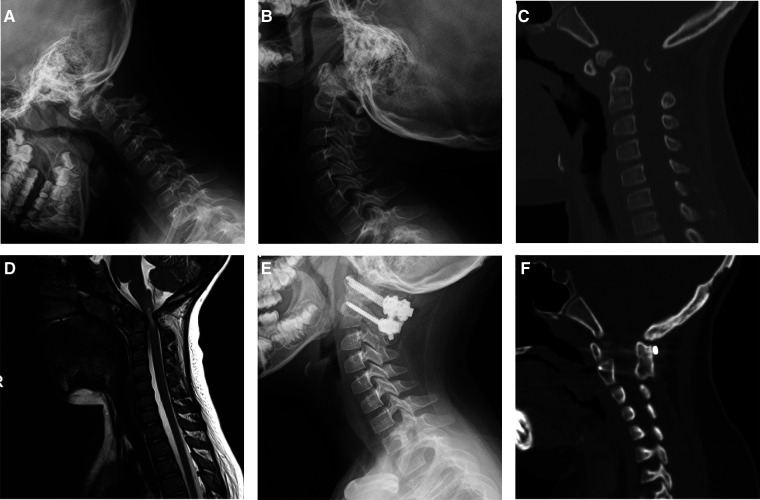
Images of a 8-year-old boy with atlantoaxial dislocation due to os odontoideum. This patient underwent atlantoaxial fusion with the use of OBG. Preoperative plain radiograph in flexion (**A**), extension (**B**) showed atlantoaxial dislocation. CT showed os odontoideum (**C**). MRI showed neural element compression (**D**). Postoperative plain radiograph in neutral (**E**) showed rigid fixation. Computed tomography sagittal reconstruction showed solid bony fusion 12 months after surgery, white arrow showed occipital bone donor-site (**F**).

Fusion surgery with OBG or IBG was performed in 19 and 20 patients, and a summary of patient characteristics is shown in Table 2. No significant differences were observed in sex, age, height, weight, atlan-dens interval (ADI), or primary diagnosis between the two groups. The operative and postoperative data are shown in Table 3; both groups achieved a 100% bone fusion rate (Figures 2, 3). In the IBG group, the fusion time was 6.1 ± 0.7 months, significantly shorter than that in the OBG group (7.4 ± 2.1 months). Estimated blood loss was significantly lower and the length of hospitalization was significantly shorter in the OBG group than in the IBG group.

**Table 2:** Summary of patients’ characteristics.

	Occipital bone graft group	Iliac bone graft group	*P* value
Sex (male : female)	10 : 9	13 : 7	0.5231
Age (years)	7.1 ± 4.6	8.9 ± 3.5	0.2609
Height (cm)	116.3 ± 31.3	114.7 ± 23.9	0.8791
Weight (kg)	28.7 ± 17.6	24.2 ± 12.2	0.7870
ADI (mm)	5.6 ± 1.6	6.0 ± 1.0	0.4898
**Primary diagnosis**			
Os odontoideum	11	12	0.7168
Hypoplasia of the dens	6	6	
Atlantoaxial rotatory displacement	2	1	
Relaxation of the transverse ligament	0	1	

**Table 3:** Comparison of operative and postoperative data.

	Occipital bone graft group	Iliac bone graft group	*P* value
Operation time (min)	99.5 ± 24.8	145.5 ± 58.5	0.0004
Estimated blood loss (ml)	73.7 ± 25.7	127.5 ± 44.4	0.0001
Length of hospitalization (days)	7.5 ± 1.7	9.5 ± 2.1	0.0077
Sugery-related complication	1 (Cerebrospinal fluid leak)	5 (Seroma formation, Infection, Donor-site pain)	0.1818
Fusion rate (%)	100	100	0.9999
Average fusion time (months)	7.7 ± 2.1	6.1 ± 1.0	0.0067
**Fusion time after surgery**			
≤6 months	9	17	0.0296
7–9 moths	7	3	
10–12 months	3	0	

There was a higher, but not significant, complication rate in the IBG group than in the OBG group. In the OBG group, a cerebrospinal fluid leak was found in one patient during surgery. There were two cases of seroma formation, two of chronic donor-site pain, and one of donor-site infection in the IBG group. All patients with preoperative neurological deficits achieved significant relief at 6-month follow-ups.

## 4. Discussion

For atlantoaxial dislocation, the primary goal of surgery is to achieve bone fusion; however, decompressing neural elements and restoring the stability and structure of the atlantoaxial joint are also important. With the application of the C1–C2 screw/rod construct technique, which provides effective biomechanical stability, patients who underwent rigid fixation showed higher fusion rates and lower complication rates than those who underwent the wire fixation technique (21–23); however, rigid fixation alone without solid bony fusion could not provide long-term stability of the atlantoaxial joint. Thus, rigid fixation and bony fusion are required for satisfactory long-term clinical outcomes. Bone graft materials are key factors for achieving solid bony fusion. Autografts and allografts are two major bone graft materials used in clinical practice. Although the use of allografts has eliminated donor-site morbidity with a relatively high fusion rate, autologous bone grafts have always been the gold standard for posterior cervical fusion because of their osteogenic and osteoinductive advantages (24). Autografts can be harvested from the iliac crest, posterior superior iliac spine, rib, or occipital bone. For structural autografts, IBG have been widely accepted as the reference standard bone material for atlantoaxial fusion. However, iliac bone harvesting is often associated with donor-site pain, prolonged operation time, increased blood loss, and pelvic fracture (25, 26). In pediatric patients, iliac bone harvesting has the potential risk of injuring the growth plate and further affecting the growth of the iliac bone. The type of bone grafting technique also affects the bone fusion process. Morselized bone grafts have optimal osteoconductive properties for the porosity of bone grafts but provide limited structural strength and integrity (27). Structural bone grafts provide excellent mechanical strength and structural integrity. This technique has a relatively low excessive bony fusion rate and can avoid venous plexus injury. Considering the advantages of the structural bone grafting technique, we used the occipital bone as an autologous structural bone graft material to treat pediatric patients diagnosed with atlantoaxial dislocation and compared the clinical outcomes of OBG and IBG.

To harvest the OBG, the bone harvesting area was 1.5 cm × 2 cm, and the external occipital protuberance was the center of the bone harvesting area. However, pediatric patients have a thinner occipital bone than adult patients, which raises a serious safety concern for harvesting this bone from them. During the occipital bone harvesting procedure, the risk of penetrating the full thickness of the occipital bone, dural laceration, and cerebrospinal fluid leakage resulting from a thinner bone harvesting area, deserves serious consideration. To address this safety issue, we evaluated the thickness of the occipital bone harvesting area from the CT scans of 300 pediatric patients. The results showed that male and female pediatric patients aged 2–6 years had a significantly thinner occipital bone than those aged 7–12 years; the average edge thickness of the bone harvesting area was 4.5 ± 1.0 mm in the younger group. Previous studies have described the techniques of harvesting full- and split-thickness grafts from the occipital or calvarial bone (12–14, 28), one recommended that the calvarial bone grafting technique should be applied in patients aged >1.5 years; however, no study has assessed the relationship between the safety of occipital bone harvesting and the thickness of the occipital donor site. We considered that an edge thickness of the occipital donor site >4 mm was relatively safe according to our clinical experience. Consequently, we selected pediatric patients who met the criteria for occipital bone grafting, and the results showed a relatively low incidence of complications related to donor site thickness.

With the application of the rigid internal fixation technique, both groups achieved a 100% fusion rate at 12 months of follow-up. However, the fusion time in the OBG group was significantly longer than that in the IBG group. Two patients who underwent occipital bone grafting did not achieve bony fusion until 12 months postoperatively, possible reason explaining the prolonged fusion time of these patients was that most of bone grafts harvested from occipital bone were cortical. Most patients in the iliac bone graft group achieved bony fusion 6 months postoperatively. The IBG mainly consist of cancellous bone, whereas most OBGs are cortical. A cancellous IBG, containing osteoblasts and osteoinductive factors, has properties of osteogenesis, osteoconduction, and osteoinduction (27, 29). A cortical OBG possesses osteoconductive properties and provides limited osteoinductive properties (29). Furthermore, the success of bony fusion is not merely due to the function of the bone graft; the decorticated transplantation bed and morselized bone harvested from the transplantation bed are also involved in the osteogenic process. Hence, the nature of the cancellous bone graft explains the significantly shorter fusion time in the IBG group. Sheehan et al. reported an 81% overall fusion rate with the use of IBG in patients who underwent atlantoaxial fusion surgery; however, the evaluation period of fusion was 6–12 weeks, which is relatively short. The wire loop was used as the fixation method, providing less stability than the screw/rod construct (9). Casey et al. demonstrated a 100% fusion rate using OBG for posterior cervical fusion over a one-year follow-up period. However, in the patients included in that study, the wire fixation technique was applied with less stability and halo bracing. However, they used a reverse hockey-stick graft shape and the full-thickness graft to compensate for the weakness of wire fixation (14). Bauman et al. applied rigid screw/rod fixation and halo bracing for pediatric patients with occipital bone grafting, and all patients achieved bony fusion during the clinical follow-up period. Structural bone grafts were placed bilaterally between C1 and C2, which was different from that in our surgical method. Compared with the bilateral grafts, our transplantation technique using one piece of a bone graft created a more stable structure (13). In our present study, comparable fusion rates in both groups showed that the occipital bone grafting technique provided guaranteed results combined with the use of rigid internal fixation.

Compared with the OBG group, the IBG group had a significantly longer operation time, increased estimated blood loss, and prolonged length of hospitalization. The prolonged length of hospitalization in the IBG group may have resulted from the second incision and the subsequent slow recovery. Harvesting IBG resulted in injury to the cancellous bone of the ilium, followed by an increase in the estimated blood loss.

Furthermore, harvesting the iliac bone requires a second incision during surgery, whereas occipital bone grafting requires no additional incision. Previous studies have reported that donor-site morbidity ranged from 10% to 24% in pediatric patients who underwent iliac bone harvesting (30, 31). The most common donor-site complication is donor-site pain. In IBG group, two patients reported chronic donor-site pain. One patient in the IBG group experienced donor-site infection, and two had seroma postoperatively. None of the patients experienced life-threatening or severe surgery-related complications. Cerebrospinal fluid leakage was observed in one 3-year-old patient who underwent occipital bone grafting. The cause of the cerebrospinal fluid leak was an accidental breakage of the inner table of the occipital bone by the osteotome. We speculated that the child had a relatively thin occipital bone, and the subsequent CT scan confirmed our speculation. The results of the occipital thickness evaluation revealed that children aged between 2 and 6 years had a significantly thinner occipital bone than children aged between 6 and 12 years, with only 4.5 mm thickness at the edge of the donor site—the occipital donor-site defect redeveloped at the last follow-up in 13 of 20 patients. In seven patients, the outer table of the occipital donor site was not regrown at the last follow-up. The defect of the occipital donor site was treated using bone wax for bleeding control. We did not use a morselized or structural bone to repair the occipital defect. The long-lasting occipital outer table defect possibly resulted from the excessive use of bone wax or over-harvesting the diploe. As for methods of managing occipital osteotomy complications, patient diagnosed of cerebrospinal fluid leakage was first accept conservative treatment including bed rest with head elevated and the prophylactic use of antibiotics. For the defect of donor-site, limiting the use of bone wax is recommended.

Our study has some limitations. First, this was a retrospective study with a small sample size. Second, to protect pediatric patients, the starting point of routine CT scanning was 6 months postoperatively, which was insufficient to assess the fusion time accurately. According to our previous experience, some adult patients achieved bony fusion in less than 3 months postoperatively using IBG. Pediatric patients have a greater potential for osteogenesis; thus, they can achieve bony fusion within 3 months or less.

## 5. Conclusion

The greatest strength of OBG is a fusion rate comparable to that of IBG. Thus, OBG are acceptable bone grafting materials for pediatric patients with atlantoaxial dislocation and they avoids donor-site morbidity and complications.

## Data Availability

The original contributions presented in the study are included in the article/Supplementary Material, further inquiries can be directed to the corresponding authors.

## References

[1] Yang SY Boniello AJ Poorman CE Chang AL Wang S Passias PG. A review of the diagnosis and treatment of atlantoaxial dislocations. Global Spine J. (2014) 4(3):197–210. 10.1055/s-0034-1376371 25083363 PMC4111952

[2] Subin B Liu JF Marshall GJ Huang HY Ou JH Xu GZ. Transoral anterior decompression and fusion of chronic irreducible atlantoaxial dislocation with spinal cord compression. Spine. (1995) 20(11):1233–40. 10.1097/00007632-199506000-00004 7660230

[3] Fielding JW Hawkins RJ Ratzan SA. Spine fusion for atlanto-axial instability. J Bone Joint Surg Am. (1976) 58(3):400–7. 10.2106/00004623-197658030-00020 1262375

[4] Wang C Yan M Zhou H Wang S Dang G. Atlantoaxial transarticular screw fixation with morselized autograft and without additional internal fixation: technical description and report of 57 cases. Spine. (2007) 32(6):643–6. 10.1097/01.brs.0000257539.75693.cc 17413468

[5] Zhang YH Shen L Shao J Chou D Song J Zhang J. Structural allograft versus autograft for instrumented atlantoaxial fusions in pediatric patients: radiologic and clinical outcomes in series of 32 patients. World Neurosurg. (2017) 105:549–56. 10.1016/j.wneu.2017.06.048 28624564

[6] Heuer GG Hardesty DA Bhowmick DA Bailey R Magge SN Storm PB. Treatment of pediatric atlantoaxial instability with traditional and modified Goel-Harms fusion constructs. Eur Spine J. (2009) 18(6):884–92. 10.1007/s00586-009-0969-x 19357876 PMC2899651

[7] Murphy RF Glotzbecker MP Hresko MT Hedequist D. Allograft bone use in pediatric subaxial cervical spine fusions. J Pediatr Orthop. (2017) 37(2):e140–e4. 10.1097/BPO.0000000000000691 26600298

[8] Reintjes SL Amankwah EK Rodriguez LF Carey CC Tuite GF. Allograft versus autograft for pediatric posterior cervical and occipito-cervical fusion: a systematic review of factors affecting fusion rates. J Neurosurg Pediatr. (2016) 17(2):187–202. 10.3171/2015.6.PEDS1562 26496632

[9] Sheehan JM Jane JA. Occipital bone graft for atlantoaxial fusion. Acta Neurochir (Wien). (2000) 142(6):661–6; discussion 7. 10.1007/s007010070110 10949441

[10] Goodrich JT Argamaso R Hall CD. Split-thickness bone grafts in complex craniofacial reconstructions. Pediatr Neurosurg. (1992) 18(4):195–201. 10.1159/000120662 1472432

[11] Zins JE Whitaker LA. Membranous versus endochondral bone: implications for craniofacial reconstruction. Plast Reconstr Surg. (1983) 72(6):778–85. 10.1097/00006534-198312000-00005 6196801

[12] Chadduck WM Boop FA. Use of full-thickness calvarial bone grafts for cervical spinal fusions in pediatric patients. Pediatr Neurosurg. (1994) 20(1):107–12. 10.1159/000120773 8142276

[13] Bauman JA Hardesty DA Heuer GG Storm PB. Use of occipital bone graft in pediatric posterior cervical fusion: an alternative paramedian technique and review of the literature. J Neurosurg Pediatr. (2011) 7(5):475–81. 10.3171/2011.2.PEDS10331 21529187

[14] Casey AT Hayward RD Harkness WF Crockard HA. The use of autologous skull bone grafts for posterior fusion of the upper cervical spine in children. Spine. (1995) 20(20):2217–20. 10.1097/00007632-199510001-00007 8545715

[15] Srivastava SK Aggarwal RA Nemade PS Bhosale SK. Single-stage anterior release and posterior instrumented fusion for irreducible atlantoaxial dislocation with basilar invagination. Spine J. (2016) 16(1):1–9. 10.1016/j.spinee.2015.09.037 26409417

[16] Yeom JS Kafle D Nguyen NQ Noh W Park KW Chang BS Routine insertion of the lateral mass screw via the posterior arch for C1 fixation: feasibility and related complications. Spine J. (2012) 12(6):476–83. 10.1016/j.spinee.2012.06.010 22795381

[17] Thomas JA Tredway T Fessler RG Sandhu FA. An alternate method for placement of C-1 screws. J Neurosurg Spine. (2010) 12(4):337–41. 10.3171/2009.10.SPINE08541 20367368

[18] Nockels RP Shaffrey CI Kanter AS Azeem S York JE. Occipitocervical fusion with rigid internal fixation: long-term follow-up data in 69 patients. J Neurosurg Spine. (2007) 7(2):117–23. 10.3171/SPI-07/08/117 17688049

[19] Harms J Melcher RP. Posterior C1–C2 fusion with polyaxial screw and rod fixation. Spine. (2001) 26(22):2467–71. 10.1097/00007632-200111150-00014 11707712

[20] Hillard VH Fassett DR Finn MA Apfelbaum RI. Use of allograft bone for posterior C1-2 fusion. J Neurosurg Spine. (2009) 11(4):396–401. 10.3171/2009.5.SPINE08662 19929334

[21] Hwang SW Gressot LV Rangel-Castilla L Whitehead WE Curry DJ Bollo RJ Outcomes of instrumented fusion in the pediatric cervical spine. J Neurosurg Spine. (2012) 17(5):397–409. 10.3171/2012.8.SPINE12770 22998404

[22] Ma C Wu J Zhao M Dai W Wu D Wang Z Treatment of upper cervical spine instability with posterior fusion plus atlantoaxial pedicle screw. Cell Biochem Biophys. (2014) 69(3):693–7. 10.1007/s12013-014-9854-2 24687596

[23] Ma X Yin Q Xia H Wu Z Yang J Liu J The application of atlantoaxial screw and rod fixation in revision operations for postoperative re-dislocation in children. Arch Orthop Trauma Surg. (2015) 135(3):313–9. 10.1007/s00402-014-2150-1 25567195

[24] Lin B Yu H Chen Z Huang Z Zhang W. Comparison of the PEEK cage and an autologous cage made from the lumbar spinous process and laminae in posterior lumbar interbody fusion. BMC Musculoskelet Disord. (2016) 17(1):374. 10.1186/s12891-016-1237-y 27577978 PMC5004315

[25] Lv C Li X Zhang H Lv J Zhang H. Comparative effectiveness of two different interbody fusion methods for transforaminal lumbar interbody fusion: cage versus morselized impacted bone grafts. BMC Musculoskelet Disord. (2015) 16:207. 10.1186/s12891-015-0675-2 26285579 PMC4545367

[26] Szadkowski M Bahroun S Aleksic I Vande Kerckhove M Ramos-Pascual S Saffarini M Bioactive glass grants equivalent fusion compared to autologous iliac crest bone for ALIF: a within-patient comparative study. J Exp Orthop. (2022) 9(1):56. 10.1186/s40634-022-00496-6 35713816 PMC9206065

[27] Baldwin P Li DJ Auston DA Mir HS Yoon RS Koval KJ. Autograft, allograft, and bone graft substitutes: clinical evidence and indications for use in the setting of orthopaedic trauma surgery. J Orthop Trauma. (2019) 33(4):203–13. 10.1097/BOT.0000000000001420 30633080

[28] Haque A Price AV Sklar FH Swift DM Weprin BE Sacco DJ. Screw fixation of the upper cervical spine in the pediatric population. Clinical article. J Neurosurg Pediatr. (2009) 3(6):529–33. 10.3171/2009.2.PEDS08149 19485741

[29] Finkemeier CG. Bone-grafting and bone-graft substitutes. J Bone Joint Surg Am. (2002) 84(3):454–64. 10.2106/00004623-200203000-00020 11886919

[30] Kager AN Marks M Bastrom T Newton PO. Morbidity of iliac crest bone graft harvesting in adolescent deformity surgery. J Pediatr Orthop. (2006) 26(1):132–4. 10.1097/01.bpo.0000188996.36674.56 16439918

[31] Skaggs DL Samuelson MA Hale JM Kay RM Tolo VT. Complications of posterior iliac crest bone grafting in spine surgery in children. Spine. (2000) 25(18):2400–2. 10.1097/00007632-200009150-00021 10984795

